# Prevalence of Electronic Cigarette Use in Saudi Arabia

**DOI:** 10.7759/cureus.25731

**Published:** 2022-06-07

**Authors:** Nawaf K Althobaiti, Mohammad Eid M Mahfouz

**Affiliations:** 1 Taif University Faculty of Medicine, Taif University, Taif, SAU

**Keywords:** electronic cigarettes' e-cigarettes' vaping' e-smoking

## Abstract

Background: The electronic cigarette (e-cigarette) was aggressively promoted as a healthier alternative to tobacco smoking. Despite the fact that e-cigarettes gained popularity among youth and young adults, there are no national data assessing the prevalence of e-cigarette use in Saudi Arabia.

Objective: The present study aims at establishing the prevalence and correlates of electronic cigarette use among adults in Saudi Arabia.

Design and setting: A cross-sectional study was carried out to evaluate the prevalence of using electronic cigarettes in Saudi Arabia through a self-administered questionnaire. The data collected from the questionnaire were focusing on socio-demographic data as well as information on the type of smoking that adults are using. Data were represented in terms of frequencies and valid percentages for categorical variables. Statistical analysis was carried out using SPSS version 26.

Results: A total of 3374 participants responded to this questionnaire. Around 26% of this cohort have tried electronic cigarettes at least once in their lives. Additionally, there was a statistically significant difference (p value <0.001) in using smokeless tobacco, where e-cigarette smokers had higher incidence of smokeless tobacco smoking. Also, e-cigarette smokers had significantly higher (p value=0.002) frequency of smoking tobacco. Moreover, e-cigarette smoking was significantly higher among males (p value <0.001), with higher educational levels (p value <0.001) and age group between 18 and 24 years old.

Conclusion: Electronic cigarette use is more common among young adults and those who have previously tried tobacco smoking. Other randomized studies are urgent to explore the extent of harmful effects of electronic cigarettes smoking (e-smoking) in the Kingdom.

## Introduction

In 2014, The Food and Drug Administration (FDA) defined electronic cigarettes, also known as e-cigarettes, as products that are operated by batteries and can deliver nicotine, as well as other chemicals [1]. The practice of e-cigarettes is known as vaping and it does not include burning tobacco, which makes it different from traditional smoking habits [2]. Users of an e-cigarette inhale the mist which constitutes nicotine [3].

E-cigarettes are wrongly thought to be safer than tobacco products, however, they are considered hazardous and unsafe for the respiratory system [4]. Despite this fact, it is increasingly used and promoted globally, particularly among young adults, even in families with high social and financial levels [5]. Unfortunately, the media influences the increasing use of e-smoking by claiming that they are less harmful in comparison to other smoking modes [6].

Additionally, young adults are attracted to e-cigarettes due to the rumors of being helpful to stop tobacco smoking [7]. This could be because younger adults become attracted to and influences by new trends especially if they think that new trend would help them get rid of an unhealthy habit which is tobacco smoking. Also, the availability of a wide range of flavors makes them preferred by most of the users [8]. Furthermore, smokers who cannot quit smoking believe that e-cigarettes are a better option with lower health hazards [9].

Another important danger of e-cigarettes is their risk of cancer [10]. Recent reports have shown that the chemical analysis of e-cigarettes exhibited to have a variety of carcinogens that raises the risk of cancer [11]. This could be added to the risk of respiratory diseases including asthma and chronic obstructive pulmonary disease, in addition to the hazards of passive smoking [12].

Despite the data available on the hazards of tobacco smoking and e-cigarettes, there is a scarcity of data on the prevalence of using e-cigarettes in Saudi Arabia and the factors contributing to their increased use in the Kingdom [13].

Therefore, the aim of this study is to establish the prevalence of e-cigarettes in Saudi Arabia and describing the correlates and reasons for smoking e-cigarettes.

## Materials and methods

Study design

A cross-sectional study to evaluate the prevalence of using electronic cigarettes and their types among the Saudi population using a self-administered designed questionnaire. Only completed surveys were included.

Data collection

Data was collected through the responses to the questionnaire. Data collectors distributed the questionnaire between November and December 2019. The study included Saudis aged 15 years and more. The questions included information on the responder’s sociodemographic data, in addition to questions to evaluate the prevalence of e-cigarette smoking, reasons for smoking, and frequency of using e-cigarettes.

Statistical analysis

All data were recorded in a pre-designed and validated excel sheet. Data were represented in terms of frequencies and valid percentages for categorical variables. Data were analyzed using IBM SPSS (Statistical Package for the Social Science; IBM Corp, Armonk, NY, USA) to perform all statistical calculations, version 26 for Microsoft Windows.

Ethical considerations

Institutional research ethics board approval was obtained from Taif University before conducting any study procedure (approval 41-710-0016). Participant identity was kept confidential.

## Results

Socio-demographic variables

This study included 3374 participants who responded to this survey. 44.3% of the responders were females, while 55.7% were males. 97.6% of the participants were Saudi. As for ages, 59.3% were between 18 and 24 years old, while only 0.6% were 65 years old or above.

Also, 65.5% had a university degree, while 1.7% had an elementary school degree. 58.4% of responders had a monthly income between 3000 to 6000 Riyals, and 63.7% were single. All socio-demographic data are detailed in Table 1.

**Table 1:** Socio-demographic characteristics of the participants

	Frequency	Percent
Gender		
Male	1878	55.7
Female	1496	44.3
Nationality		
Non-Saudi	80	2.4
Saudi	3294	97.6
Age		
15 - 17	156	4.6
18 - 24	2000	59.3
25 - 34	795	23.6
35 - 44	275	8.2
45 - 64	128	3.8
65 or over	20	0.6
Educational level		
Elementary	58	1.7
Mid school	211	6.3
High school	894	26.5
University graduate or above	2211	65.5
Monthly income		
3000 - 6000	1969	58.4
7000 - 10000	844	25.0
11000 - 15000	280	8.3
More than 15000	281	8.3
Marital status		
Single	2148	63.7
Married	1148	34.0
Divorced	56	1.7
Widowed	22	0.7
Employment status		
Student	1831	54.3
Public	980	29.0
Private	238	7.1
Housewife	245	7.3
Retired	80	2.4
	Frequency	Percent
Gender		
Male	1878	55.7
Female	1496	44.3
Nationality		
Non-Saudi	80	2.4
Saudi	3294	97.6
Age		
15 - 17	156	4.6
18 - 24	2000	59.3
25 - 34	795	23.6
35 - 44	275	8.2
45 - 64	128	3.8
65 or over	20	0.6
Educational level		
Elementary	58	1.7
Mid school	211	6.3
High school	894	26.5
University graduate or above	2211	65.5
Monthly income		
3000 - 6000	1969	58.4
7000 - 10000	844	25.0
11000 - 15000	280	8.3
More than 15000	281	8.3
Marital status		
Single	2148	63.7
Married	1148	34.0
Divorced	56	1.7
Widowed	22	0.7
Employment status		
Student	1831	54.3
Public	980	29.0
Private	238	7.1
Housewife	245	7.3
Retired	80	2.4

**Figure 1:**
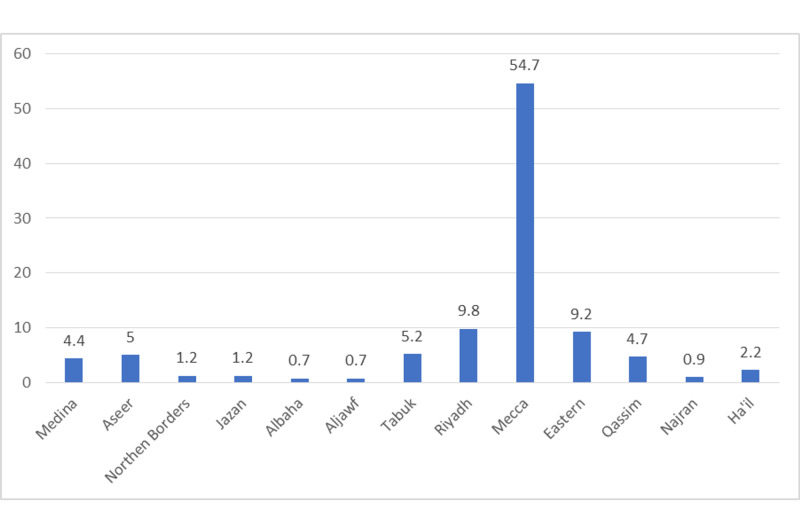
Place of residence of the participants

Participants were also asked about their place of residence. It was found that 54.7% of the responders were from Mecca province, while 0.7% were from Albaha or Aljawf province as shown in Figure 1.

Participants were also asked about smoking tobacco and e-cigarettes. 26.3% smoked e-cigarettes at least once and 24.7% had a family member who is an e-smoker. Also, 8.2% used smokeless tobacco and 18.2% smoked more than 100 tobacco cigarettes in their lifetime, with 50.5% having a family member who smoked tobacco as shown in Table 2.

**Table 2:** Smoking tobacco and e-cigarettes

Questions		Frequency	Percent
Have you ever tried electronic cigarettes (e-cigarettes) even once?	No	2485	73.7
	Yes	889	26.3
If you are an e-cigarettes smoker, do you have any e-cigarettes smoker in your family?	No	1022	75.3
	Yes	336	24.7
Have you ever had use smokeless tobacco products even once?	No	3099	91.8
	Yes	275	8.2
Have you ever smoked 100 tobacco cigarettes in your lifetime?	No	2759	81.8
	Yes	615	18.2
If you are a tobacco smoker, do you have any tobacco smoker in your family	No	511	49.5
	Yes	521	50.5

Additionally, responders were asked about their reasons for using electronic cigarettes. 19.5% wanted to quit smoking tobacco cigarettes, while 16.2% thought they are safer compared to tobacco cigarettes. 35.3% had other reasons as shown in Table 3.

**Table 3:** Reasons for using electronic cigarettes

Reasons	Frequency	Percent
Wanted to quit smoking cigarettes	231	19.5
Safer than tobacco cigarettes?	192	16.2
Recommendation	115	9.7
Wanted to replace smoking cigarettes some of the time	101	8.5
Cheaper than tobacco cigarettes	65	5.5
Wanted to smoke in places where cigarettes smoking is not allowed	61	5.2
Others	417	35.3

Participants also were asked about their frequency of using tobacco and e-cigarettes. 6.9% used e-cigarettes on daily basis, while 10.6% used tobacco cigarettes every day as shown in Table 4.

**Table 4:** Frequency of using electronic cigarettes and tobacco cigarettes

Questions		Frequency	Percent
Do you use e-cigarettes now?	Everyday	239	6.9
	Some days	212	6.1
	No	3024	87.0
Do you smoke tobacco cigarettes now?	Everyday	369	10.6
	Some days	187	5.4
	No	2918	84.0

As for responders who had quit smoking, 62.3% quit smoking more than one year and less than three years, while 11.8% quit smoking in less than one year as shown in Table 5.

**Table 5:** Duration since quitting smoking

Duration since quitting smoking	Frequency	Percent
More than one year and three years or less	401	62.3
Less than one year	76	11.8
More than three years	167	25.9

Finally, different variables were compared over responders who had tried e-cigarettes and those who had not using the chi-square test at level of significance p value < 0.05.

The comparison revealed that there is a statistically significant difference (p value <0.001) in using smokeless tobacco, where e-cigarette smokers had higher incidence of smokeless tobacco use. Also, they had significantly higher (p value=0.002) frequency of smoking tobacco.

Moreover, e-cigarette smoking was significantly higher among males (p value <0.001), with higher educational levels (p value <0.001) and age group between 18 and 24 years old as shown in Table 6.

**Table 6:** Comparison of different variables between e-cigarette smokers and non-smokers

Parameters		Did not try e-cigarettes	Tried e-cigarettes	P value
Have you ever used smokeless tobacco products even once?	No	96.9%	77.8%	<0.001*
	Yes	3.1%	22.2%	
Do you smoke tobacco cigarettes now?	Everyday	9.8%	12.7%	0.002*
	Some days	4.9%	7.0%	
	No	85.3%	80.3%	
Gender	Female	68.0%	21.1%	<0.001*
	Male	32.0%	78.9%	
Educational level	Elementary school	1.9%	1.1%	<0.001*
	Intermediate	8.0%	1.3%	
	High school	29.0%	19.5%	
	University	61.0%	78.1%	
Age	15 - 17	5.5%	2.1%	<0.001*
	18 - 24	56.5%	66.9%	
	25 - 34	24.0%	22.3%	
	35 - 44	9.0%	5.8%	
	45 - 64	4.3%	2.2%	
	65 or over	0.6%	0.6%	

## Discussion

E-cigarette use is currently increasing in both developing and developed countries among young adults [14]. It has been mistakenly thought that it is a safer option compared to tobacco smoking. However, recent reports have shown that e-cigarettes can have similar hazards to those imposed by tobacco smoking [15]. Hence, it is important to understand the features of e-cigarettes in our nations.

The aim of the present work is to explore the prevalence e-cigarette use in Saudi Arabia. It is revealed that 26.3% of this cohort have tried e-cigarettes at least once in their lives. Additionally, there was a statistically significant difference (p value <0.001) in using smokeless tobacco, where e-cigarette smokers had higher incidence of smokeless tobacco smoking.

Also, e-cigarette smokers had significantly higher (p value=0.002) frequency of smoking tobacco. Moreover, e-cigarette smoking was significantly higher among males (p value <0.001), with higher educational levels (p value <0.001) and age group between 18 and 24 years old.

Prevalence of e-cigarette use has been investigated in different settings. Czoli et al. [16] investigated the prevalence of e-cigarette use in Canada among young adults. 1188 participants responded to a questionnaire on using e-cigarettes and revealed that the awareness of young adult Canadians on using e-cigarettes is relatively high with a 16.1% prevalence of e-cigarette use [16].

In the present study, the prevalence of using e-cigarettes was higher (26.3%) compared to the Canadian population. Additionally, e-smoking in Saudi Arabia has been linked to male gender, high educational level, smoking tobacco and higher smoking frequency.

Another study from Poland by Goniewicz et al. evaluated the use of e-cigarettes among teenagers and young adults. Through a survey analysis that included 20240 responders, they showed that about one-fifth of the Polish population smoke e-cigarettes. The study also recommended increasing the awareness of young adults on the hazards of e-smoking [17].

The present study also demonstrated that the prevalence of e-cigarette smoking is almost one-fifth of the included cohort. Moreover, the present study also examined the correlates to e-cigarette smoking that were not examined by either Czoli et al. [16] or Goniewicz et al. [17]. This makes the current study more informative in this regard. 

These findings were also supported by Dockrell et al. [18] in the United Kingdom who also demonstrated that e-cigarettes were more prevalent among people who had a previous history of tobacco smoking.

To our knowledge, this is the first study to evaluate the prevalence of e-cigarettes in Saudi Arabia. However, the present study had some limitations that may question the outcomes. The study depended mainly on the honesty of the responder while responding to these questions to identify the prevalence of using e-cigarettes. Therefore, other randomized controlled trials to identify the prevalence and risk factors of the disease are mandatory.

## Conclusions

E-cigarette use is more common among young adults and those who have previously tried tobacco smoking. Other randomized studies are urgent to explore the extent of harmful effects of e-smoking in the Kingdom. These findings should be considered by healthcare professionals in Saudi Arabia to improve the awareness of the Saudi community towards the hazards of e-cigarette smoking.

## Appendices

Q1: Sex

1 - Male

2- Female

Q2: Age

1-15-17

2- 18-24

3- 25-34

4- 35-44

5- 45-64

6- 65 or above

Q3- Nationality

1-Saudi

2-Non-saudi

Q4- Educational level :

1-Less than high school

2-High school graduate

3-Associate degree

4-College graduate and above

Q5-Monthly Income level:

1-Less than 5000 SR

2-5000-14000

3-15000-24000

4- 25000 and above

Q6- Marital Status:

1-Never married

2-Married spouse present

3-Widowed/Divorced/Separated

Q7- Residency:

1-Rural

2-Urban

Q8- Employment status:

1-Public sector employee.

2-Private sector employee.

3-BussinesMan/Woman.

4-Non-Employee.

Q9- Have you ever tried electronic cigarettes (e-cigarettes) even once ?

1-Yes.

2-No.

If Yes, please answer the next four questions

Why did you try using an electronic cigarettes ?

1-Wanted to quit smoking cigarettes.

2-Wanted to replace smoking cigarettes some of the time.

3-Wanted to smoke in places where cigarettes smoking is not allowed.

4-Cheaper than tobacco cigarettes.

5-Safer than tobacco cigarettes

6-Recommendation

7-Others

Which of the brands have ever used ?

1-Choose from 15 brands list.

2-Other.

3-Don't know.

 Q10-Do you use it now

1-Everyday.

2-Somedays.

3-Not at all.

If you use e-cigarettes some days, how many of the past 30 days did you use an e-cigarettes ?

1-Once.

2-20 or more days.

Which brands are you currently using ?

1-Choose from 15 brands list.

2-Other.

3-Don't know.

If you are an e-cigarettes smoker, do you have any e-cigarettes smoker in your family ?

1-Yes.

2-No.

If yes, have they started smoking e-cigarettes after or before you being an e-cigarettes smoker ?

1-After.

2-Before.

Q11- Have you ever had use smokeless tobacco products Even once?

1-Yes

2-No

If yes,do you use smokeless tobacco products now?

1-Everyday

2-some days

3-Not at all

If you use it some days, how many  of the past 30 days did you use smokeless tobacco products ?

1-Once.

2- 20 or more days.

Q12-Have you ever smoke 100 tobacco cigarettes in your lifetime ?

1-Yes

2-No

Q13- Do you smoke tobacco cigarettes now ?

1-Everyday.

2-Some days.

3-Not at all.

If you chose choice 2, how many times did you smoke in the last month ?

 (open question) or putting 30 choice

Q14- If you are smoker, when do you smoke your first cigarette after been awake ? 

1-Less than 5 minutes.

2-5-30 minutes.

3-More than 30 minutes.

If you are a tobacco smoker, do you have any tobacco smoker in your family ?

1-Yes.

2-No.

If yes, have they started smoking tobacco after or before you being a tobacco cigarettes smoker ?

1-After.

2-Before.

Q15- If you smoked 100 tobacco cigarettes in your lifetime and quit smoking now , how long have you been quite smoking ?

1-One year or less.

2-More than one year and three years or less.

3-More than three years.
